# Supplementary material for: Epistatic Relationship between MGV1 and TRI6 in the Regulation of Biosynthetic Gene Clusters in Fusarium graminearum
Source: J Fungi (Basel). 2023 Aug 2;9(8):816. doi: 10.3390/jof9080816 (PMC10455978; doi:10.3390/jof9080816)
Supplement: Supplementary file 1 [file jof-09-00816-s001.zip › Supplementary Figures_Final.pptx]

## Slide 1
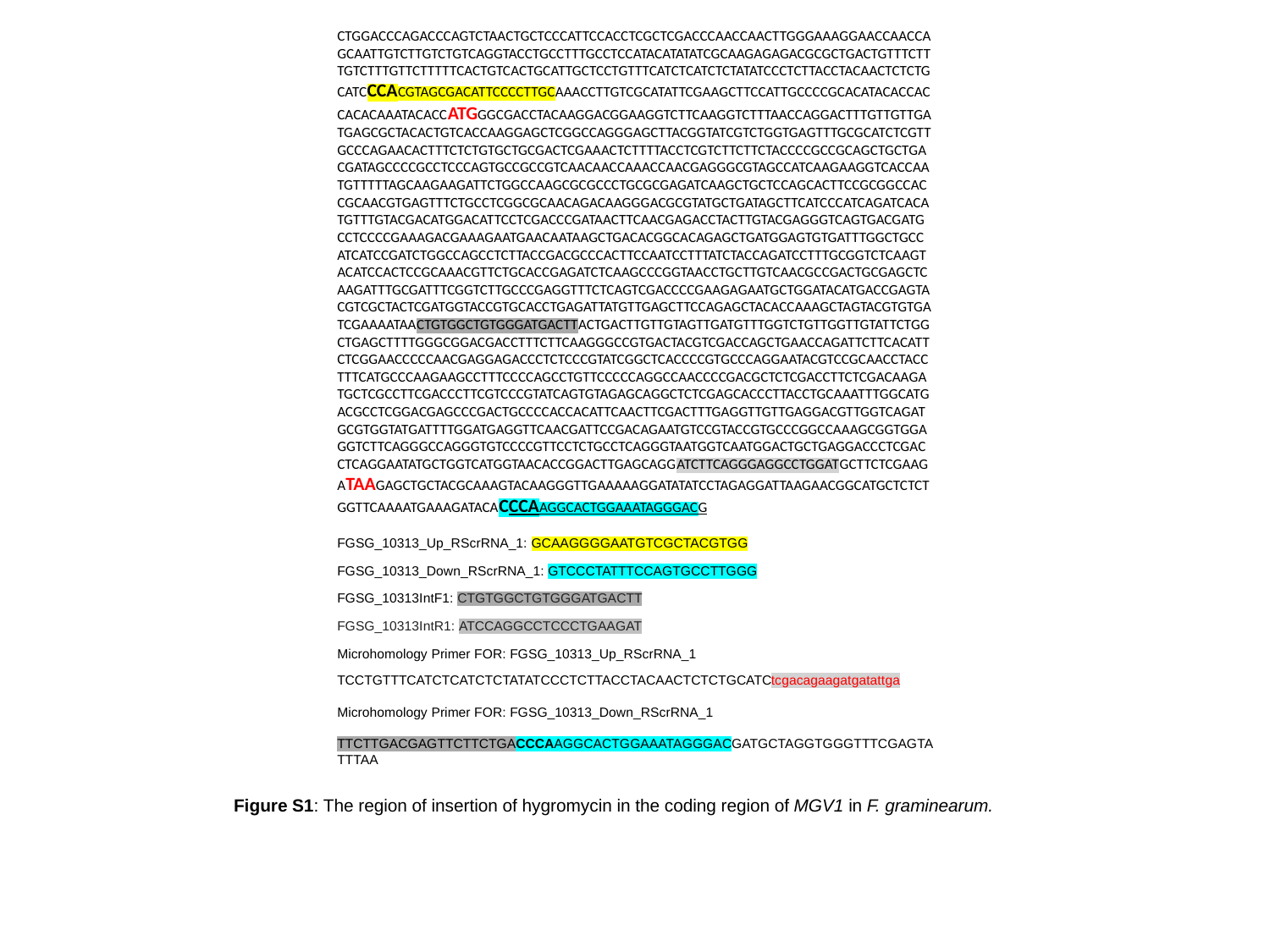

CTGGACCCAGACCCAGTCTAACTGCTCCCATTCCACCTCGCTCGACCCAACCAACTTGGGAAAGGAACCAACCAGCAATTGTCTTGTCTGTCAGGTACCTGCCTTTGCCTCCATACATATATCGCAAGAGAGACGCGCTGACTGTTTCTTTGTCTTTGTTCTTTTTCACTGTCACTGCATTGCTCCTGTTTCATCTCATCTCTATATCCCTCTTACCTACAACTCTCTGCATCCCACGTAGCGACATTCCCCTTGCAAACCTTGTCGCATATTCGAAGCTTCCATTGCCCCGCACATACACCACCACACAAATACACCATGGGCGACCTACAAGGACGGAAGGTCTTCAAGGTCTTTAACCAGGACTTTGTTGTTGATGAGCGCTACACTGTCACCAAGGAGCTCGGCCAGGGAGCTTACGGTATCGTCTGGTGAGTTTGCGCATCTCGTTGCCCAGAACACTTTCTCTGTGCTGCGACTCGAAACTCTTTTACCTCGTCTTCTTCTACCCCGCCGCAGCTGCTGACGATAGCCCCGCCTCCCAGTGCCGCCGTCAACAACCAAACCAACGAGGGCGTAGCCATCAAGAAGGTCACCAATGTTTTTAGCAAGAAGATTCTGGCCAAGCGCGCCCTGCGCGAGATCAAGCTGCTCCAGCACTTCCGCGGCCACCGCAACGTGAGTTTCTGCCTCGGCGCAACAGACAAGGGACGCGTATGCTGATAGCTTCATCCCATCAGATCACATGTTTGTACGACATGGACATTCCTCGACCCGATAACTTCAACGAGACCTACTTGTACGAGGGTCAGTGACGATGCCTCCCCGAAAGACGAAAGAATGAACAATAAGCTGACACGGCACAGAGCTGATGGAGTGTGATTTGGCTGCCATCATCCGATCTGGCCAGCCTCTTACCGACGCCCACTTCCAATCCTTTATCTACCAGATCCTTTGCGGTCTCAAGTACATCCACTCCGCAAACGTTCTGCACCGAGATCTCAAGCCCGGTAACCTGCTTGTCAACGCCGACTGCGAGCTCAAGATTTGCGATTTCGGTCTTGCCCGAGGTTTCTCAGTCGACCCCGAAGAGAATGCTGGATACATGACCGAGTACGTCGCTACTCGATGGTACCGTGCACCTGAGATTATGTTGAGCTTCCAGAGCTACACCAAAGCTAGTACGTGTGATCGAAAATAACTGTGGCTGTGGGATGACTTACTGACTTGTTGTAGTTGATGTTTGGTCTGTTGGTTGTATTCTGGCTGAGCTTTTGGGCGGACGACCTTTCTTCAAGGGCCGTGACTACGTCGACCAGCTGAACCAGATTCTTCACATTCTCGGAACCCCCAACGAGGAGACCCTCTCCCGTATCGGCTCACCCCGTGCCCAGGAATACGTCCGCAACCTACCTTTCATGCCCAAGAAGCCTTTCCCCAGCCTGTTCCCCCAGGCCAACCCCGACGCTCTCGACCTTCTCGACAAGATGCTCGCCTTCGACCCTTCGTCCCGTATCAGTGTAGAGCAGGCTCTCGAGCACCCTTACCTGCAAATTTGGCATGACGCCTCGGACGAGCCCGACTGCCCCACCACATTCAACTTCGACTTTGAGGTTGTTGAGGACGTTGGTCAGATGCGTGGTATGATTTTGGATGAGGTTCAACGATTCCGACAGAATGTCCGTACCGTGCCCGGCCAAAGCGGTGGAGGTCTTCAGGGCCAGGGTGTCCCCGTTCCTCTGCCTCAGGGTAATGGTCAATGGACTGCTGAGGACCCTCGACCTCAGGAATATGCTGGTCATGGTAACACCGGACTTGAGCAGGATCTTCAGGGAGGCCTGGATGCTTCTCGAAGATAAGAGCTGCTACGCAAAGTACAAGGGTTGAAAAAGGATATATCCTAGAGGATTAAGAACGGCATGCTCTCTGGTTCAAAATGAAAGATACACCCAAGGCACTGGAAATAGGGACG
FGSG_10313_Up_RScrRNA_1: GCAAGGGGAATGTCGCTACGTGG
FGSG_10313_Down_RScrRNA_1: GTCCCTATTTCCAGTGCCTTGGG
FGSG_10313IntF1: CTGTGGCTGTGGGATGACTT
FGSG_10313IntR1: ATCCAGGCCTCCCTGAAGAT
Microhomology Primer FOR: FGSG_10313_Up_RScrRNA_1
TCCTGTTTCATCTCATCTCTATATCCCTCTTACCTACAACTCTCTGCATCtcgacagaagatgatattga
Microhomology Primer FOR: FGSG_10313_Down_RScrRNA_1
TTCTTGACGAGTTCTTCTGACCCAAGGCACTGGAAATAGGGACGATGCTAGGTGGGTTTCGAGTATTTAA
Figure S1: The region of insertion of hygromycin in the coding region of MGV1 in F. graminearum.

## Slide 2
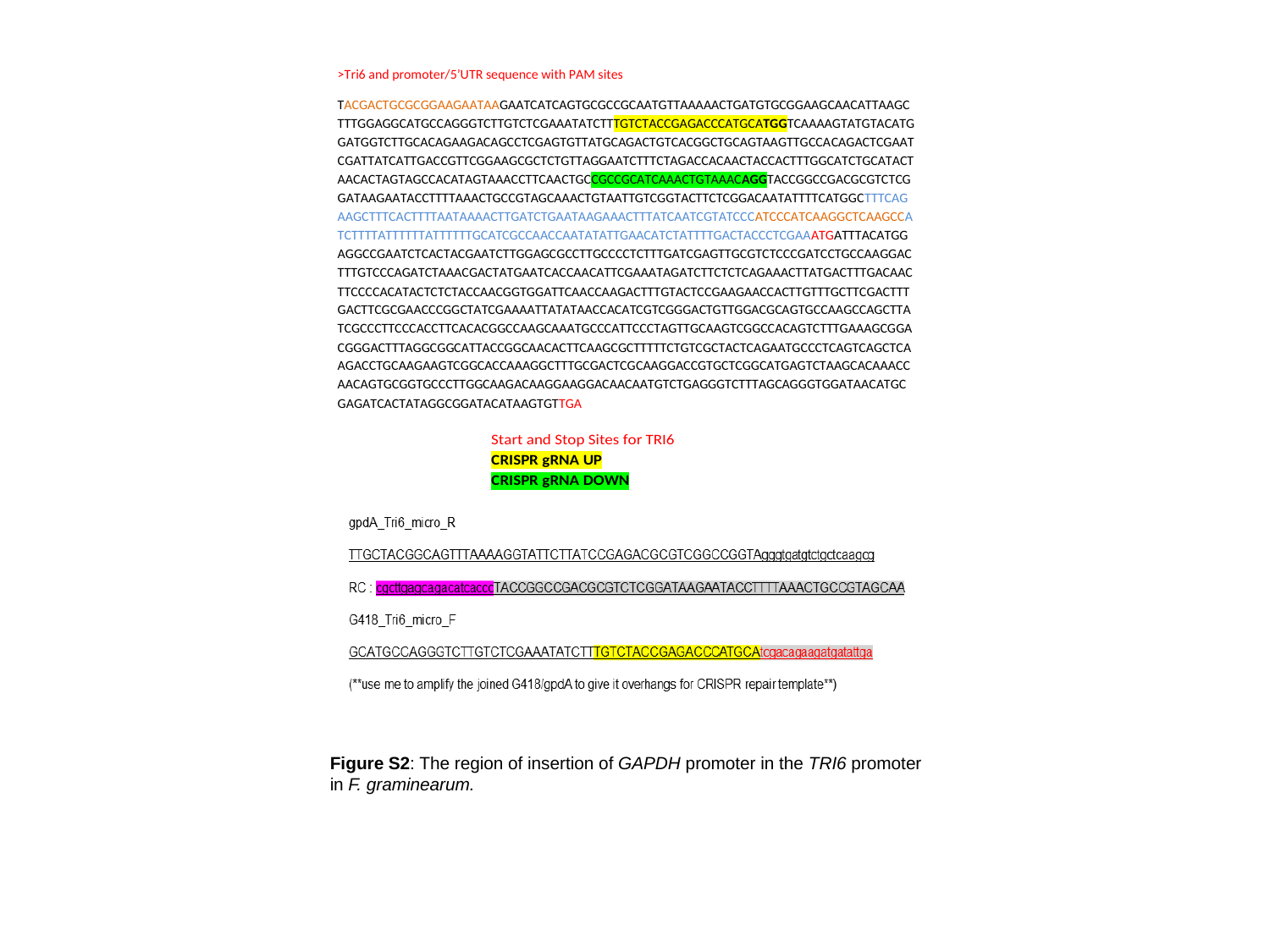

Figure S2: The region of insertion of GAPDH promoter in the TRI6 promoter in F. graminearum.

## Slide 3
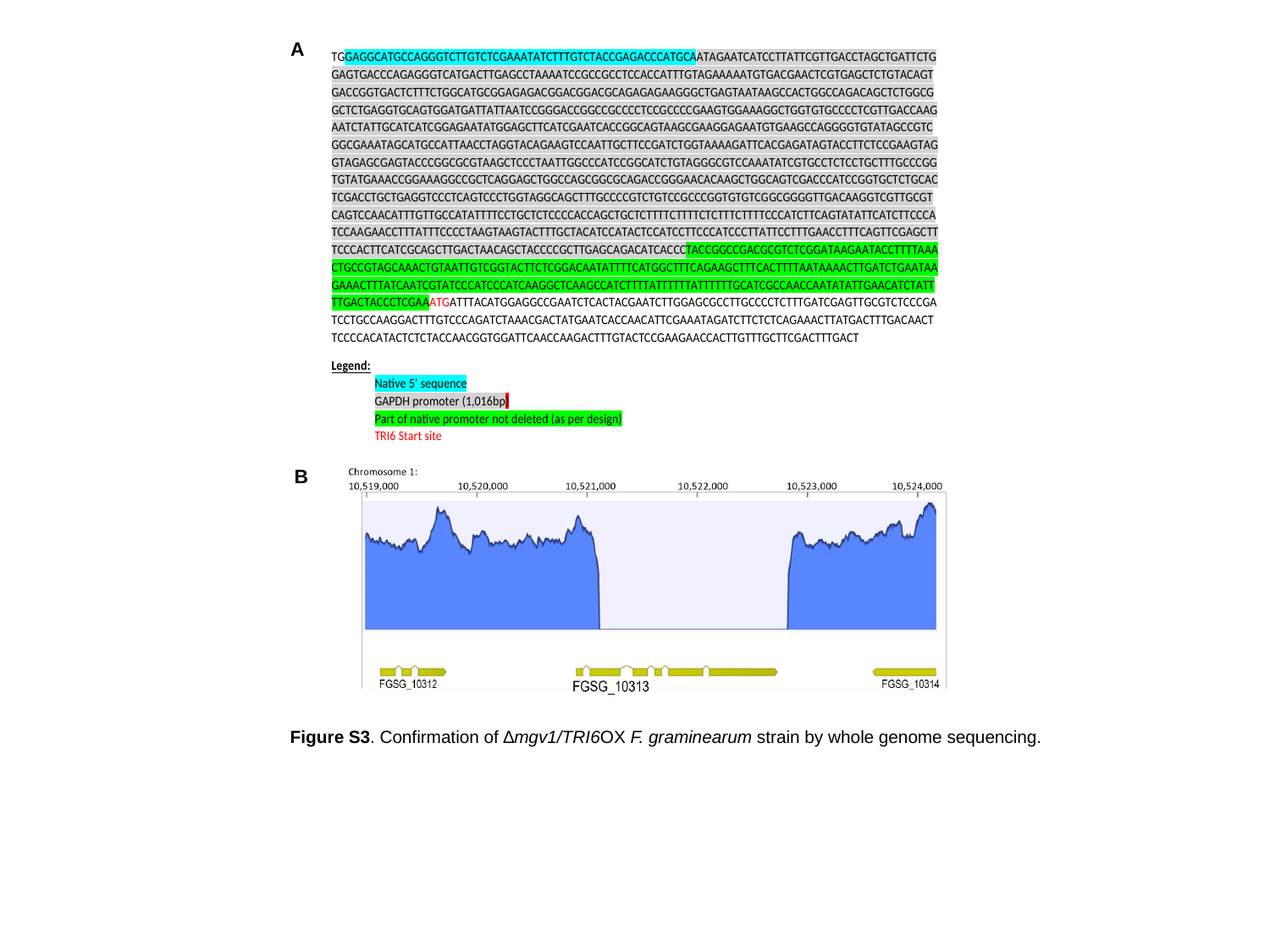

A
B
Figure S3. Confirmation of ∆mgv1/TRI6OX F. graminearum strain by whole genome sequencing.

## Slide 4
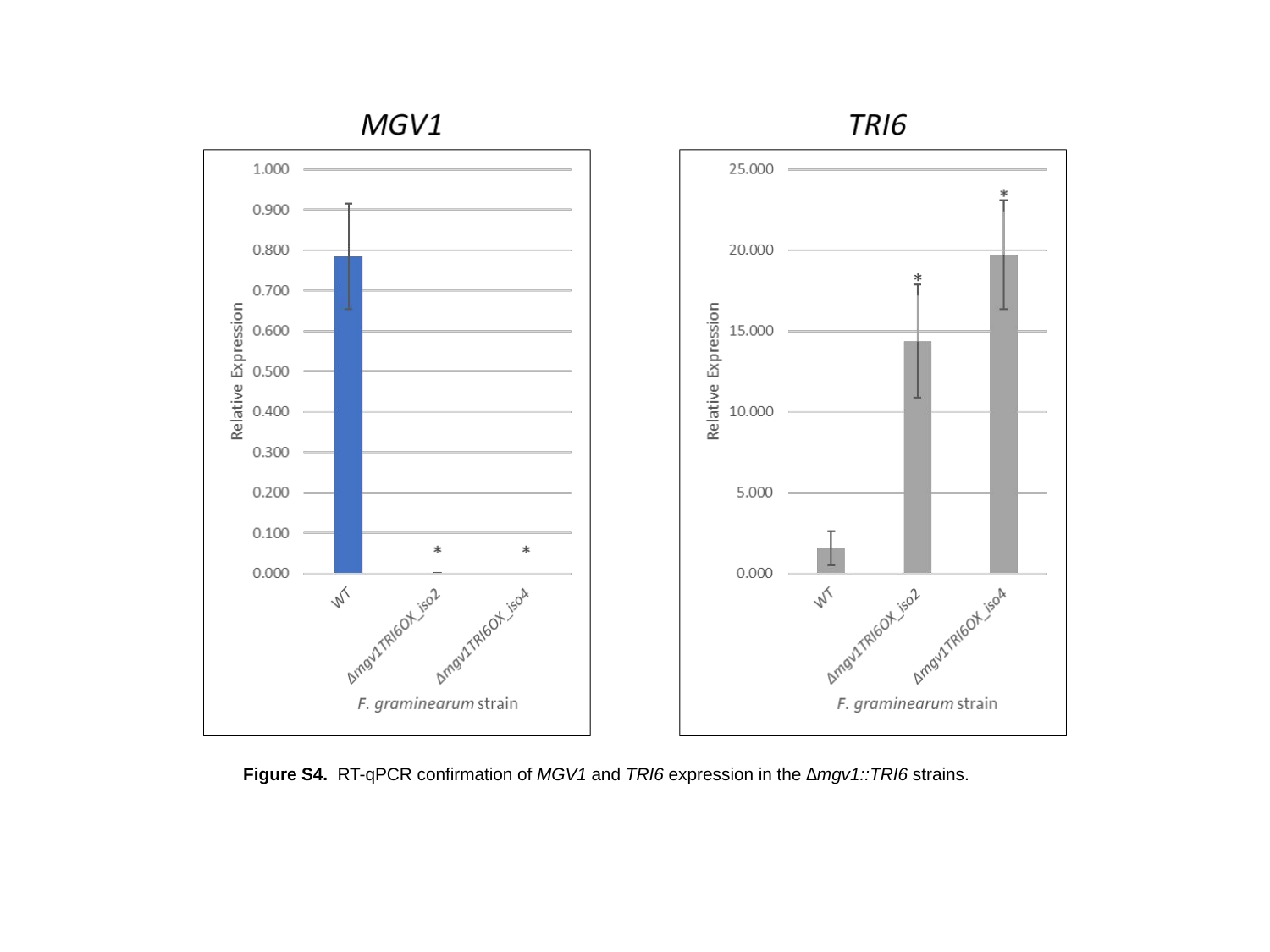

Figure S4. RT-qPCR confirmation of MGV1 and TRI6 expression in the ∆mgv1::TRI6 strains.

## Slide 5
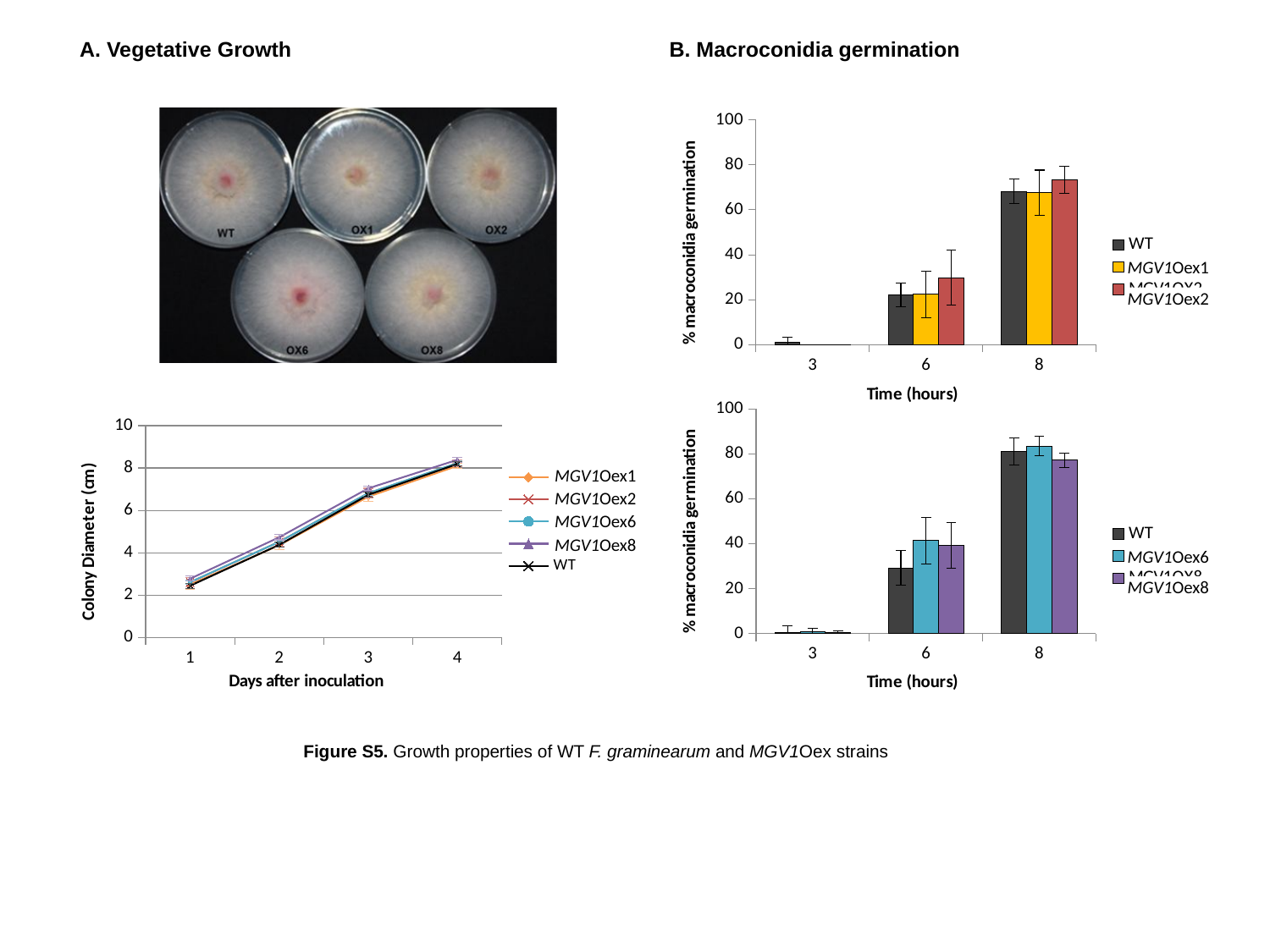

A. Vegetative Growth
B. Macroconidia germination
### Chart
| Category | | | |
|---|---|---|---|
| 3 | 0.8928571428571429 | 0.0 | 0.0 |
| 6 | 22.087247606666114 | 22.343679712100766 | 29.710962278403887 |
| 8 | 68.20270372901952 | 67.64129529096634 | 73.41633733691246 |
MGV1Oex1
MGV1Oex2
### Chart
| Category | | | | | |
|---|---|---|---|---|---|
### Chart
| Category | | | |
|---|---|---|---|
| 3 | 0.5747126436781609 | 0.7638888888888888 | 0.33783783783783783 |
| 6 | 29.13244211741654 | 41.29568924822256 | 39.13252781236069 |
| 8 | 81.10635034022131 | 83.36770619619084 | 77.10094415597133 |MGV1Oex1
MGV1Oex2
MGV1Oex6
MGV1Oex8
MGV1Oex6
MGV1Oex8
Figure S5. Growth properties of WT F. graminearum and MGV1Oex strains

## Slide 6
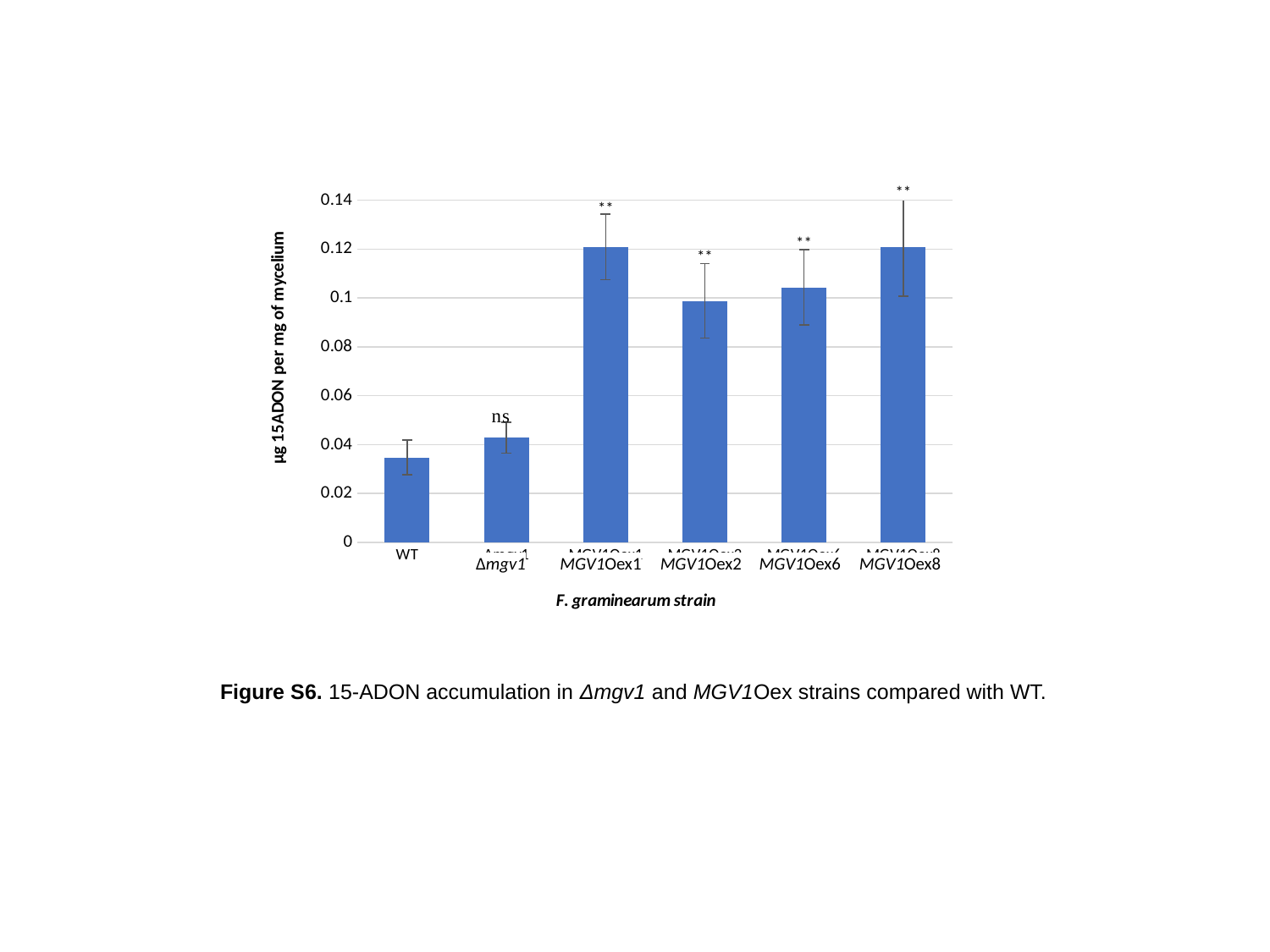

### Chart
| Category | |
|---|---|
| WT | 0.03474312672030623 |
| Δmgv1 | 0.04279201943399995 |
| MGV1Oex1 | 0.1209006927155567 |
| MGV1Oex2 | 0.09880339109811394 |
| MGV1Oex6 | 0.10436040841818345 |
| MGV1Oex8 | 0.12080280573438075 |Δmgv1
MGV1Oex1
MGV1Oex2
MGV1Oex6
MGV1Oex8
Figure S6. 15-ADON accumulation in Δmgv1 and MGV1Oex strains compared with WT.

## Slide 7
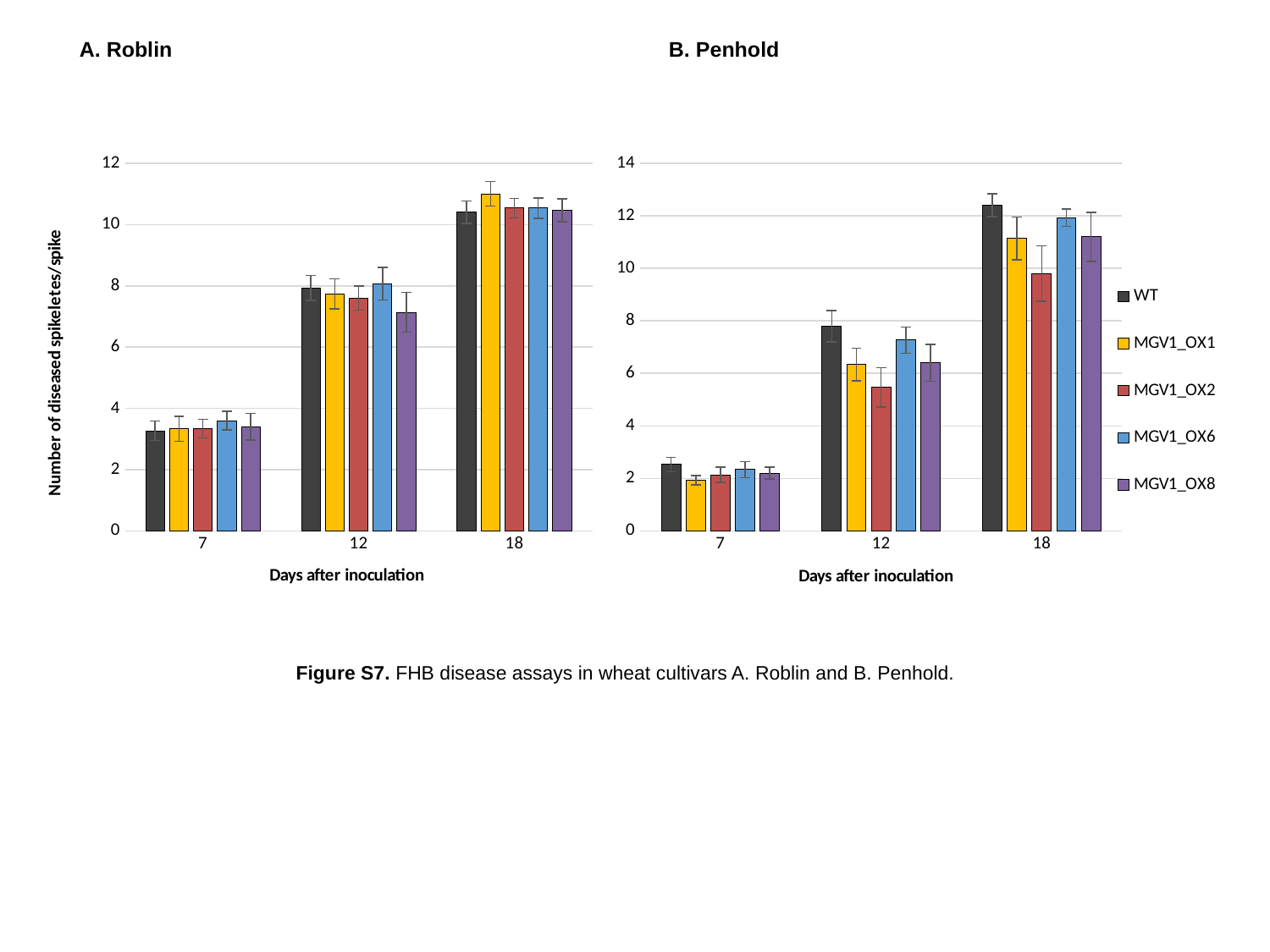

A. Roblin
B. Penhold
### Chart
| Category | WT | MGV1Oex1 | MGV1Oex2 | MGV1Oex3 | MGV1Oex4 |
|---|---|---|---|---|---|
| 7 | 3.2666666666666666 | 3.3333333333333335 | 3.3333333333333335 | 3.6 | 3.4 |
| 12 | 7.933333333333334 | 7.733333333333333 | 7.6 | 8.066666666666666 | 7.133333333333334 |
| 18 | 10.4 | 11.0 | 10.533333333333333 | 10.533333333333333 | 10.466666666666667 |
### Chart
| Category | WT | MGV1_OX1 | MGV1_OX2 | MGV1_OX6 | MGV1_OX8 |
|---|---|---|---|---|---|
| 7 | 2.533333333333333 | 1.9333333333333333 | 2.1333333333333333 | 2.3333333333333335 | 2.2 |
| 12 | 7.8 | 6.333333333333333 | 5.466666666666667 | 7.266666666666667 | 6.4 |
| 18 | 12.4 | 11.133333333333333 | 9.8 | 11.933333333333334 | 11.2 |Figure S7. FHB disease assays in wheat cultivars A. Roblin and B. Penhold.

## Slide 8
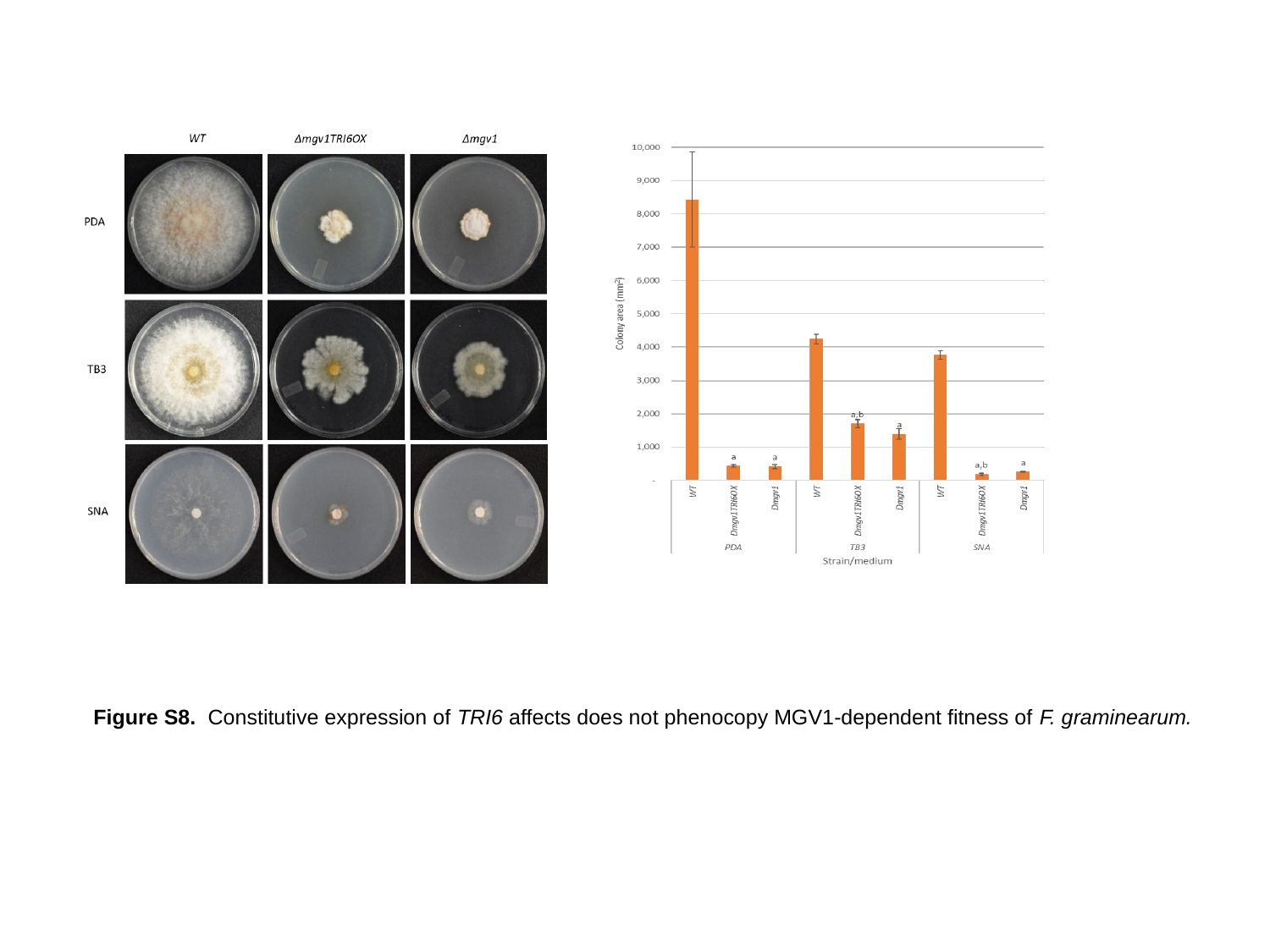

Figure S8. Constitutive expression of TRI6 affects does not phenocopy MGV1-dependent fitness of F. graminearum.
